# Propranolol Attenuates Late Sodium Current in a Long QT Syndrome Type 3-Human Induced Pluripotent Stem Cell Model

**DOI:** 10.3389/fcell.2020.00761

**Published:** 2020-08-13

**Authors:** Sayako Hirose, Takeru Makiyama, Dario Melgari, Yuta Yamamoto, Yimin Wuriyanghai, Fumika Yokoi, Suguru Nishiuchi, Takeshi Harita, Mamoru Hayano, Hirohiko Kohjitani, Jingshan Gao, Asami Kashiwa, Misato Nishikawa, Jie Wu, Jun Yoshimoto, Kazuhisa Chonabayashi, Seiko Ohno, Yoshinori Yoshida, Minoru Horie, Takeshi Kimura

**Affiliations:** ^1^Department of Cardiovascular Medicine, Kyoto University Graduate School of Medicine, Kyoto, Japan; ^2^Department of Cardiovascular and Respiratory Medicine, Shiga University of Medical Science, Otsu, Japan; ^3^Institute of Cardiometabolism and Nutrition, Sorbonne University, Paris, France; ^4^Department of Bioscience and Genetics, National Cerebral and Cardiovascular Center, Suita, Japan; ^5^Center for iPS Cell Research and Application (CiRA), Institute for Integrated Cell-Material Sciences, Kyoto University, Kyoto, Japan; ^6^Department of Pharmacology, Medical School of Xi’an Jiaotong University, Xi’an, China; ^7^Department of Cardiology, Shizuoka Children’s Hospital, Shizuoka, Japan; ^8^Center for Epidemiologic Research in Asia, Shiga University of Medical Science, Otsu, Japan

**Keywords:** long QT syndrome type 3, induced pluripotent stem cell, sodium channel, β blocker, arrhythmia

## Abstract

**Background:**

Long QT syndrome type 3 (LQT3) is caused by gain-of-function mutations in the *SCN5A* gene, which encodes the α subunit of the cardiac voltage-gated sodium channel. LQT3 patients present bradycardia and lethal arrhythmias during rest or sleep. Further, the efficacy of β-blockers, the drug used for their treatment, is uncertain. Recently, a large multicenter LQT3 cohort study demonstrated that β-blocker therapy reduced the risk of life-threatening cardiac events in female patients; however, the detailed mechanism of action remains unclear.

**Objectives:**

This study aimed to establish LQT3-human induced pluripotent stem cells (hiPSCs) and to investigate the effect of propranolol in this model.

**Method:**

An hiPSCs cell line was established from peripheral blood mononuclear cells of a boy with LQT3 carrying the *SCN5A*-N1774D mutation. He had suffered from repetitive torsades de pointes (TdPs) with QT prolongation since birth (QTc 680 ms), which were effectively treated with propranolol, as it suppressed lethal arrhythmias. Furthermore, hiPSCs were differentiated into cardiomyocytes (CMs), on which electrophysiological functional assays were performed using the patch-clamp method.

**Results:**

N1774D-hiPSC-CMs exhibited significantly prolonged action potential durations (APDs) in comparison to those of the control cells (N1774D: 440 ± 37 ms vs. control: 272 ± 22 ms; at 1 Hz pacing; *p* < 0.01). Furthermore, N1774D-hiPSC-CMs presented gain-of-function features: a hyperpolarized shift of steady-state activation and increased late sodium current compared to those of the control cells. 5 μM propranolol shortened APDs and inhibited late sodium current in N1774D-hiPSC-CMs, but did not significantly affect in the control cells. In addition, even in the presence of intrapipette guanosine diphosphate βs (GDPβs), an inhibitor of G proteins, propranolol reduced late sodium current in N1774D cells. Therefore, these results suggested a unique inhibitory effect of propranolol on late sodium current unrelated to β-adrenergic receptor block in N1774D-hiPSC-CMs.

**Conclusion:**

We successfully recapitulated the clinical phenotype of LQT3 using patient-derived hiPSC-CMs and determined that the mechanism, by which propranolol inhibited the late sodium current, was independent of β-adrenergic receptor signaling pathway.

## Introduction

Congenital long QT syndrome (LQT) is an inherited arrhythmogenic disease, associated with lethal arrhythmic events and sudden cardiac death. Patients with LQT are currently classified into over 15 genetic subtypes, and LQT1-3 accounts for approximately 90% of the genotyped patients, specifically: 40–55%, 30–45%, and 5–10% are LQT1, LQT2, and LQT3, respectively (Schwartz et al., 2012; Mizusawa et al., 2014). LQT1 and LQT2 are caused by loss-of-function mutations in *KCNQ1* and *KCNH2* genes, which encode cardiac slowly (I_Ks_) and rapidly (I_Kr_) activating delayed rectifier potassium channels, respectively (Moss et al., 2007; Shimizu et al., 2009). On the other hand, LQT3 is caused by gain-of function mutations in the *SCN5A* gene, which encodes cardiac voltage-gated sodium channels (Bennett et al., 1995).

A number of studies reported genotype-phenotype correlations among those three major genotypes (Moss et al., 2000; Schwartz et al., 2001; Priori et al., 2004; Hobbs et al., 2006), and genetic testing for patients with LQT is highly recommended for identifying carriers in the families and determining the appropriate choice of gene-specific treatment (Ackerman et al., 2011; Skinner et al., 2019). Patients with LQT1 and LQT2 suffer from cardiac events that occur during exercise or emotional stress (Schwartz et al., 2001, 2012). They are usually treated with β-blockers, as they are highly effective in reducing cardiac event and mortality rates (Schwartz et al., 2001; Priori et al., 2004). By contrast, patients with LQT3 experience cardiac events during rest or sleep (Schwartz et al., 2001, 2012), and β-blocker therapy has resulted less effective or even harmful in preventing those cardiac events, according to small cohort studies and the clinical features of LQT3 (Moss et al., 2000; Schwartz et al., 2001; Priori et al., 2004). Recently, a large clinical cohort study, which consisted of 406 patients with LQT3, demonstrated that β-blocker therapy reduced the number of cardiac events in female patients >1 year old (Wilde et al., 2016), thus β-blocker therapy has regained importance as an optimal medicine for LQT3. However, the pharmacological mechanism by which β-blockers benefit LQT3 patients is still unclear.

In the present study, we investigated the cellular mechanism by which β-blockers affected late sodium currents in human LQT3 cardiomyocytes (CMs). To this end, we established a human induced pluripotent stem cell line (hiPSCs) from a young male carrying *SCN5A*-N1774D genotype and whose repetitive torsades de pointes (TdPs) were effectively treated with propranolol. In N1774D-hiPSC-derived CMs, prolonged action potential durations (APDs) and increased late sodium current at baseline were attenuated after propranolol administration. More importantly, we determined that late sodium current inhibition by propranolol was unrelated to β-adrenergic receptor signaling pathway in the LQT3 hiPSC-CM model.

## Materials and Methods

### Generation and Characterization of LQT3-hiPSCs

We collected peripheral blood mononuclear cells from a patient carrying *SCN5A*-N1774D after obtaining the written informed consent, and employed an integration-free method using episomal vectors to generate hiPSCs (Okita et al., 2013). We used three different patient-derived iPSCs clones in this study. As control hiPSCs lines, we used two different lines (201B7 and 253G1) generated from a healthy donor (Takahashi et al., 2007; Nakagawa et al., 2008). This study was approved by the Kyoto University ethics review broad and conformed to the principles of the Declaration of Helsinki.

The pluripotency of established LQT3-hiPSCs were assessed using immunostaining and teratoma assay (Yamamoto et al., 2017; Wuriyanghai et al., 2018). Briefly, the hiPSC colonies were fixed with 4% paraformaldehyde for 20 min at 4°C. The cells were permeabilized in 0.2% Triton X-100 (Nacalai Tesque, Kyoto, Japan) and blocked with 5% FBS. The samples were stained overnight at 4°C with the following primary antibodies: mouse monoclonal anti-OCT3/4 (1:50; Santa Cruz Biotechnology, Delaware, CA, United States), mouse monoclonal anti-SSEA4 (1:200; Santa Cruz Biotechnology), and mouse monoclonal anti-TRA 1–60 (1:200; Santa Cruz Biotechnology). The secondary antibody was donkey anti-mouse Alexa fluor 488 (1:1000, Invitrogen, Carlsbad, CA, United States). The nuclei were stained with DAPI (1:2000, Wako Pure Chemical Industries, Osaka, Japan). The specimens were observed under a fluorescence microscope (Biozero BZ-9000; KEYENCE, Osaka, Japan). For teratoma assay, the hiPSCs were injected into severe combined immunodeficiency disease scid/scid mice under the testis capsule. Tumor samples were surgically dissected at 8 weeks, fixed in 10% formalin and stained with hematoxylin and eosin. All animal experiments were performed in accordance with the “Guide for the Care and Use of Laboratory Animals” (2011) of the National Institutes of Health and the Regulation on Animal Experimentation at Kyoto University, and approved by Ethics Committee of Kyoto University.

### Cardiomyocyte Differentiation

Cardiomyocyte differentiation was induced with an embryoid body (EB) differentiation system as previously described (Yang et al., 2008; Wuriyanghai et al., 2018). The hiPSCs aggregated to form EBs and cultured in suspension for 20 days. On the 20th day of culture, EBs were dispersed into small clusters using collagenase B (Roche Diagnostics GmbH, Mannheim, Germany) and trypsin EDTA (Nacalai Tesque) and were plated onto 0.1% gelatin coated dishes. After day 20, hiPSC-CMs were maintained in DMEM/F12 with 2% fetal bovine serum, 2 mmol/L L-glutamine, 0.1 mmol/L non-essential amino acids, 0.1 mmol/L β-mercaptoethanol, 50 U/ml penicillin, and 50 μg/ml streptomycin. The medium was renewed every 2–3 days. Differentiated 6–8-week-old hiPSC-CMs were enzymatically dispersed into single cells using collagenase B and trypsin EDTA. The cells were plated on 0.1% gelatin coated glass cover slips. Patch-clamp experiments were performed 4–7 days after the procedures. Cardiac differentiation was performed more than five times with each hiPSCs clone and the data pooled from different lines or clones among the control and N1774D group were analyzed.

### Electrophysiological Assay

Electrophysiological assays were performed using the whole-cell patch-clamp technique with the Multiclamp 700B microelectrode amplifier and Axon Digidata 1440 digitizer hardware (Molecular Devices, San Jose, CA, United States), as described previously (Ma et al., 2011; Hayano et al., 2017; Wuriyanghai et al., 2018).

Action potentials (APs) were recorded with perforated patch-clamp in the current mode at a bath temperature of 36 ± 1°C. APs were evoked at a constant pacing rate of 1 Hz, with 5 ms depolarizing current injections of 50–200 pA. The external solution contained (in mM): 140 NaCl, 5.4 KCl, 1.8 CaCl_2_, 1.0 MgCl_2_, 10 HEPES, 10 Glucose, pH 7.40 (adjusted with NaOH) and pipette solution contained (in mM): 150 KCl, 5 EGTA, 5 MgATP, 10 HEPES, 5 NaCl, 2 CaCl_2_ with pH of 7.2 and 300–500 μg/ml amphotericin B. The patch pipettes had a resistance of 3–6 MΩ. Ventricular-type action potentials were defined by the morphology of APs and the classification based on previous report (Matsa et al., 2011): a deep diastolic membrane potential (<−50 mV), a sharp systolic depolarization, a long plateau phase, and AP duration at 90% repolarization (action potential duration: APD_90_)/APD at 50% repolarization (APD_50_) ratio <1.4.

Sodium current was recorded at the bath temperature of 22–23°C using whole-cell voltage-clamp technique. Pipette resistances were between 0.8 and 1.8 MΩ, with access resistances of <5 MΩ. The extracellular bath solution contained (in mM): 137 NaCl, 100 TEA-Cl, 1.8 CaCl_2_, 2 MgCl_2_, 10 HEPES, 10 Glucose, 0.2 NiCl_2_, 0.005 Nifedipine (pH was adjusted to 7.4 with CsOH). The intrapipette solution was contained (in mM): 5 NaCl, 70 CsCl, 40 CSAs, 2.0 CaCl_2_, 10 EGTA, 10 HEPES, 5 MgATP, pH 7.3 (adjusted with C_s_OH). The standard holding potential was −100 mV. Each protocol in detail is illustrated in the inset.

The current-voltage relationships were fit with the Boltzmann equation: *I* = (*V*−*V*_rev_) × *G*_max_ × [1 + exp (*V*−*V*_1__/__2_)/*k*]^–1^, where *I* is the peak sodium current during the test pulse potential *V*. The parameters estimated by the fitting are reversal potential (*V*_rev_), maximum conductance (*G*_max_), and slop factor (*k*). Steady-state availability was fit to the Boltzmann equation: *I*/*I*_max_ = [1 + exp (*V*−*V*_1__/__2_)/*k*]^–1^ to determine the membrane potential for *V*_1__/__2_ (half-maximal inactivation) and *k* (slop factor). *I*_max_ is the maximum peak sodium current. Data for the time course of recovery from inactivation were fitted by a biexponential equation: *I(t)*/*I*_max_ = A_f_ [1−exp(−*t*/*τ*_f_)] + A_s_ [1−exp(−*t*/*τ*_s_)], where A_f_ and A_s_ represent fractions of each components, respectively. Time course of entry into the slow inactivation state and development of closed-state inactivation were fitted with a single exponential equation: *I*/*I*_max_ = y0 + A[1−exp(−*t*/*τ*)], where the transfer rate of sodium channels from closed-state to inactivated closed-state without an intervening opening state was measured by a double pulse protocol. Time course for development of closed-state inactivation was fit with a single exponential equation, *I*/*I*_max_ = y0 + A [1−exp(−*t*/*τ*)].

Late sodium currents were recorded for 800 ms at −10 mV before and after 20 μM tetrodotoxin (TTX; Nacalai Tesque) application. The pipette solution contained (in mM): 5 NaCl, 70 CsCl, 40 C_S_Asp, 10 EGTA, 10 HEPES, 5 MgATP, pH 7.3 (adjusted with CsOH).

To assess the effect of propranolol for APD and sodium current, recordings were performed before and after the administration of 5 μM propranolol hydrochloride (FUJIFILM Wako, Osaka, Japan).

### Statistics

Data are expressed as mean ± the standard error of measurement. Statistical comparisons were analyzed using appropriate student’s *t*-test and paired *t*-tests. A *p* value of <0.05 was considered statistically significant.

## Results

### Clinical Patient Profile

The proband was a 1-day-old infant who exhibited severe bradycardia at 27 weeks of gestation (Kato et al., 2014). His surface electrocardiogram showed an extremely prolonged QT interval (QTc 680 ms) with functional atrio-ventricular block and repeated TdPs immediately after his birth. He was suspected of long QT syndrome and thus propranolol was administered to suppress TdPs. But the treatment was not enough to suppress the electrical storm. Therefore, mexiletine was added and, as a result, TdPs disappeared.

Additionally, genetic testing identified a heterozygous *SCN5A* mutation, named p. N1774D, confirming the diagnosis of LQT3. Therefore, treatment of the patient with propranolol was discontinued. However, TdPs recurred after its interruption, and thus propranolol administration was resumed, and as a consequence, TdPs immediately disappeared.

### Generation and Characterization of *SCN5A*-N1774D-hiPSCs

**FIGURE 1:**
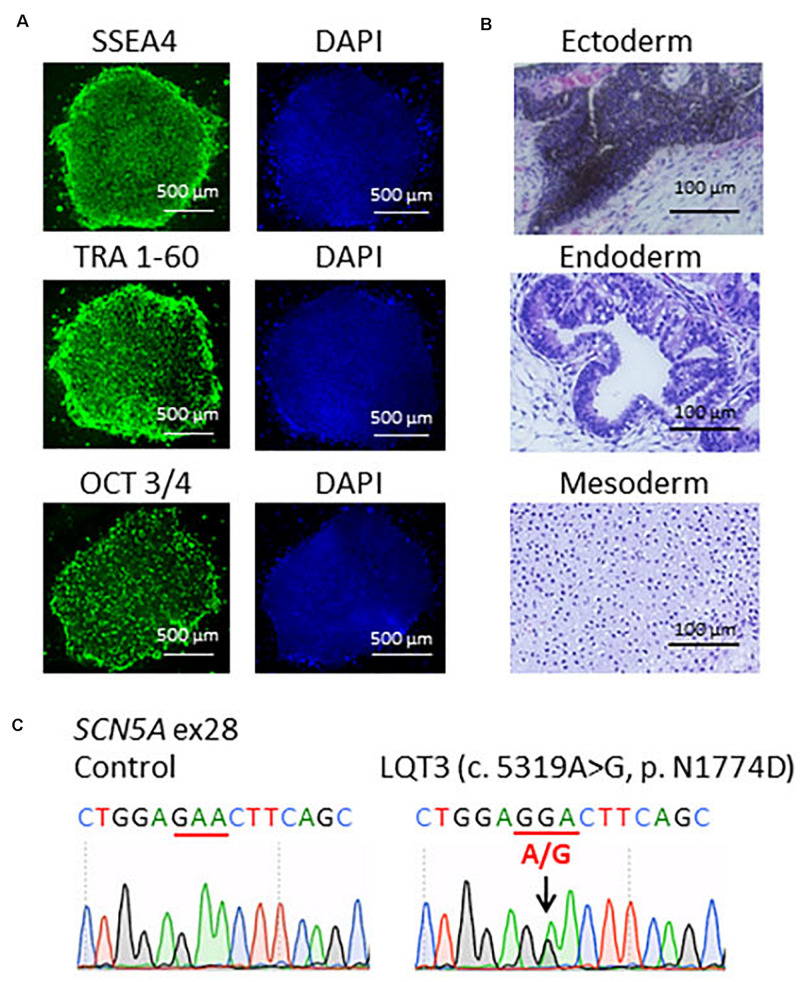
Characterization of LQT3-hiPSCs. **(A)** Immunofluorescence staining for stem cells makers. LQT3-hiPSC colonies derived from the peripheral blood mononuclear cells of a patient with *SCN5A*-N1774D expressed pluripotency markers; SSEA4, TRA 1-60, and OCT 3/4. Blue (right) showed 40-6-diamidino-2-phenylindole (DAPI) staining of nuclei. Scale bars = 500 μm. **(B)** Hematoxylin-eosin staining of teratomas formed from LQT3-hiPSC showed differentiation of the cells into various tissue derived from all three germ layers: melanocytes (ectoderm), gut-like structures (endoderm), and cartilage tissue (mesoderm). **(C)** DNA sequences of the *SCN5A* gene identified in the control hiPSCs and LQT3 cells carrying N1774D heterozygous mutation in LQT3-hiPSCs, not control hiPSCs. Scale bars: 100 μm.

Patient-specific LQT3 hiPSCs were generated from the peripheral blood mononuclear cells of the pediatric patient carrying the *SCN5A*-N1774D mutation. *SCN5A*-N1774D-hiPSCs displayed characteristics of human embryonic stem cell morphology and expressed human pluripotency makers: OCT3/4, SSEA4, and TRA-1-60 (Figure 1A). To evaluate the pluripotency of the generated hiPSC line, teratoma formation assays were performed using scid/scid mice. The teratomas contained tissues derivatives of three germ layers: ectoderm, endoderm, and mesoderm (Figure 1B). Furthermore, the *SCN5A*-N1774D mutation (c.5319A > G) was confirmed in the patient-derived hiPSCs, but not identified in the control samples (Figure 1C).

### Prolonged APDs in N1774D-hiPSC-CMs

**FIGURE 2:**
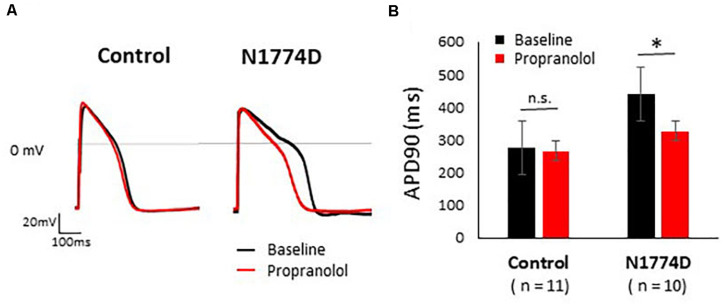
Effect of propranolol on action potential recording in control and N1774D-hiPSC-CMs. **(A)** Representative traces of paced ventricular-type action potential (AP) at 1 Hz pacing at baseline (black line) and after the administration 5 μM propranolol (red line) in control (left) and N1774D-hiPSC-CMs (right). **(B)** Summarized data in effects of propranolol on AP duration of control and N1774D-hiPSC-CMs. The data pooled from different lines or clones among the control and N1774D group were analyzed. ^∗^ *p* < 0.001, vs. control. APD_90_ was measured at 90% repolarization (APD_90_). APD_90_ values in N1774D-hiPSC-CMs were significantly prolonged compared with those in control. Propranolol significantly shortened the values of APD_90_ in N1774D-hiPSC-CMs. ^∗^ *p* < 0.001, vs. baseline.

Action potentials of hiPSC-CMs were recorded using the current-clamp technique. Figure 2A shows typical ventricular-type APs. For this study, we measured APDs at 50% and 90% repolarization, APD_50_ and APD_90_, respectively, maximum diastolic potential, and AP amplitude.

Action potential durations in N1774D-hiPSC-CMs were significantly prolonged compared to those in control hiPSC-CMs at 1 Hz pacing (APD_50_ and APD_90_: N1774D-hiPSC-CMs, 440 ± 37 ms and 377 ± 33 ms, respectively, *n* = 11, vs. control, 272 ± 22 ms and 192 ± 18 ms, respectively, *n* = 10; *p* < 0.01; Figure 2 and Table 1). However, no significant difference in other AP parameters was observed (Table 1).

**TABLE 1:** AP parameters at baseline and after administration of propranolol in control and N1774D-hiPSC-CMs at 1 Hz pacing.

	Control		N1774D	
	
	Baseline	Propranolol 5 μM	Baseline	Propranolol 5 μM
	
	(*N* = 11)	(*N* = 11)	(*N* = 10)	(*N* = 10)
RMP (mV)	−69.3 ± 2.9	−67 ± 3.0	−70.0 ± 2.4	−69.9 ± 1.5
MDP (mV)	−75.8 ± 2.9	−72.7 ± 2.7	−76.7 ± 3.3	−74.9 ± 2.2
APA (mV)	114.4 ± 3.4	114.4 ± 3.1	120.4 ± 1.6	119.9 ± 1.5
Max dV/dt (mV/ms)	30.2 ± 3.7	36.2 ± 9.0	32.8 ± 4.4	31.8 ± 3.6
APD_50_ (ms)	192 ± 18	178 ± 18^†^	377 ± 33^#^	268 ± 31^†^
APD_90_ (ms)	272 ± 22	267 ± 26	440 ± 37^#^	332 ± 37^†^

*AP, action potential; RMP, resting membrane potential: The threshold potential at which the action potential is initiated; MDP, maximum diastolic potential; APA, AP amplitude; Max dV/dt, maximum rate of rise of the AP upstroke; APD_50_ and APD_90_, AP durations at 50% and 90%. The data pooled from different lines or clones among the control and N1774D group were analyzed. The number of experiments is indicated in parentheses. Data are showed as means ± standard error of measurement. ^#^*p* < 0.01 vs. Control, ^†^*p* < 0.01 vs. Baseline.*

### N1774D-hiPSC-CMs Exhibited Increased Late Sodium Current

**FIGURE 3:**
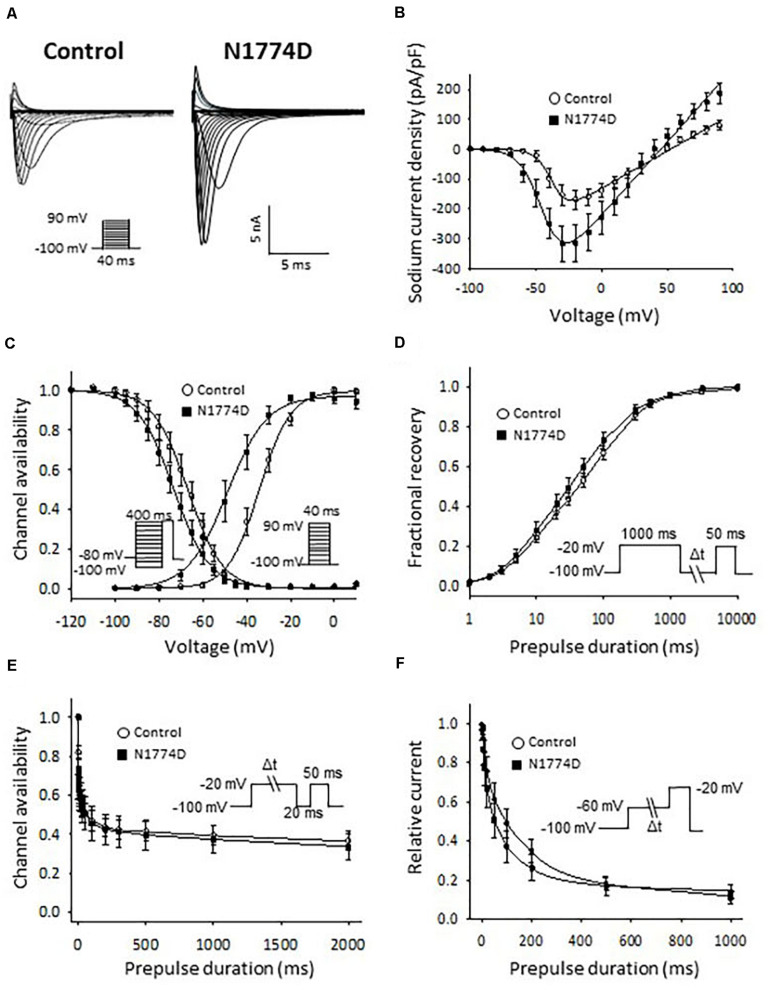
Sodium current recordings and gating properties of sodium channels in hiPSC-CMs. **(A)** Representative traces of sodium currents in control and N1774D-hiPSC-CMs. The pulse protocol is shown in the inset. **(B)** Average current-voltage relationship for peak sodium current in control (*n* = 15, open circles) and N1774D channels (*n* = 15, closed squares). Data were fitted with the Boltzmann equation (see “Materials and Methods”). The currents were normalized to the cell capacitance to give a measure of peak current densities. The peak current densities were significantly lager in N1774D. **(C)** Voltage dependence of steady-state inactivation and activation in control and N1774D-hiPSC-CMs. Curves were fit using the Boltzmann equation. The activation curve negatively shifted by 13 mV. **(D)** Time course of recovery from inactivation was obtained by a double pulse potential shown in inset. Experimental data were fit to a biexponential. **(E)** Onset of slow inactivation. Time course of entry into the slow inactivation state was measured by a double pulse protocol shown in inset. Curves were fit with a shingle exponential equation. **(F)** Closed-state inactivation. The transfer rate of sodium channels from closed-state to inactivated closed-state without an intervening opening state was elicited with a double pulse protocol shown in inset. The data pooled from different lines or clones among the control and N1774D group were analyzed.

The sodium channel currents in N1774D-hiPSC-CMs and control cells were recorded by using the voltage-clamp technique (Figure 3 and Table 2). Figure 3A illustrates representative whole-cell current traces of N1774D-hiPSC-CMs and control cells. As shown in Figure 3B, the peak sodium current densities of N1774D-hiPSC-CMs were significantly increased, compared to those of the control cells (N1774D: −333 ± 62 pA/pF, *n* = 15 vs. control: −175 ± 33 pA/pF, *n* = 15; *p* < 0.005; Table 2). Moreover, the steady-state activation of sodium channels, shifted to more negative potentials by 13 mV in N1774D-hiPSC-CMs, compared to that of the control cells (Figure 3C and Table 2). No significant differences in the voltage dependence of inactivation and other kinetic properties were noted between control cells and N1774D-hiPSC-CMs.

**TABLE 2:** Biophysical properties in control and N1774D-hiPSC-CMs.

	Control	N1774D
Peak I_Na_ density	(*N* = 15)	(*N* = 15)
	−175 ± 33	−333 ± 62^†^
Steady-state activation	(*N* = 15)	(*N* = 15)
V_1/2_	−34.9 ± 1.6	−47.7 ± 3.5^†^
*k*	5.6 ± 0.5	4.9 ± 0.3
Steady-state fast inactivation	(*N* = 11)	(*N* = 12)
V_1/2_	−67.4 ± 2.5	−73.2 ± 2.4
*k*	6.3 ± 0.2	6.7 ± 0.1
Recovery from inactivation	(*N* = 12)	(*N* = 11)
τ_f_ (ms)	21.5 ± 6.3	24.9 ± 4.8
τ_s_ (ms)	227.2 ± 42.4	236.8 ± 43.4
Onset of slow inactivation	(*N* = 11)	(*N* = 8)
A	0.47 ± 0.06	0.56 ± 0.07
τ (ms)	14.4 ± 4.0	22.9 ± 8.7
Closed-state inactivation	(*N* = 10)	(*N* = 9)
A	0.89 ± 0.02	0.84 ± 0.07
τ (ms)	123.1 ± 32.0	74.6 ± 17.4

*Data are expressed as means ± standard error of measurement. Parameters were calculated from fitting individual experiments illustrated in Figure 3. The data pooled from different lines or clones among the control and N1774D group were analyzed. ^†^*p* < 0.01 vs. Control. A, fractional amplitude; τ, time constant; V_1/2_, midpoint potential; *k*, slop factor, and *n*, number of tested cells, respectively.*

**FIGURE 4:**
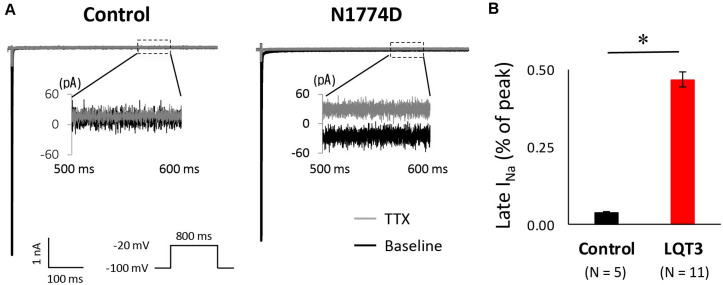
Late sodium current in control and N1774D-hiPSC-CMs. **(A)** Representative traces of sodium currents in the absence (black line) and presence (gray line) of 20 μM tetrodotoxin (TTX). The used protocol is shown in the lower panel. Inset shows late sodium current between 500 and 600 ms. Tetrodotoxin-sensitive current was calculated by subtraction. **(B)** Mean late sodium current of control and N1774D channels. Late sodium current is presented as the percentage of late sodium current to peak sodium current. The data pooled from different lines or clones among the control and N1774D group were analyzed. The late sodium current was significantly increased. ^∗^*p* < 0.001, vs. control.

Next, we measured the late sodium current for both cell lines. Figure 4A shows representative whole-cell sodium current traces from control and N1774D-hiPSC-CMs. The ratio of late/peak sodium current in N1774D-hiPSC-CMs was significantly increased compared with that in control cells (N1774D: 0.47 ± 0.04%, *n* = 11, vs. control, 0.04 ± 0.01%, *n* = 5; *p* < 0.0001; Figure 4B).

### Propranolol Shortened APDs and Reduced Late Sodium Current in N1774D-hiPSC-CMs

We assessed the effect of propranolol on APDs and late sodium current using hiPSC-CMs. Propranolol shortened APDs in N1774D-hiPSC-CMs (APD_90_: 440 ± 37 ms at baseline vs. 332 ± 37 ms after propranolol treatment, respectively, *n* = 10; at 1 Hz pacing; *p* < 0.01; Figure 2 and Table 1), but propranolol did not significantly affect APDs in the control cells (APD_90_: 272 ± 22 ms at baseline vs. 267 ± 26 ms after propranolol treatment, respectively, *n* = 11; at 1 Hz pacing; *p* = 0.15; Figure 2 and Table 1).

**FIGURE 5:**
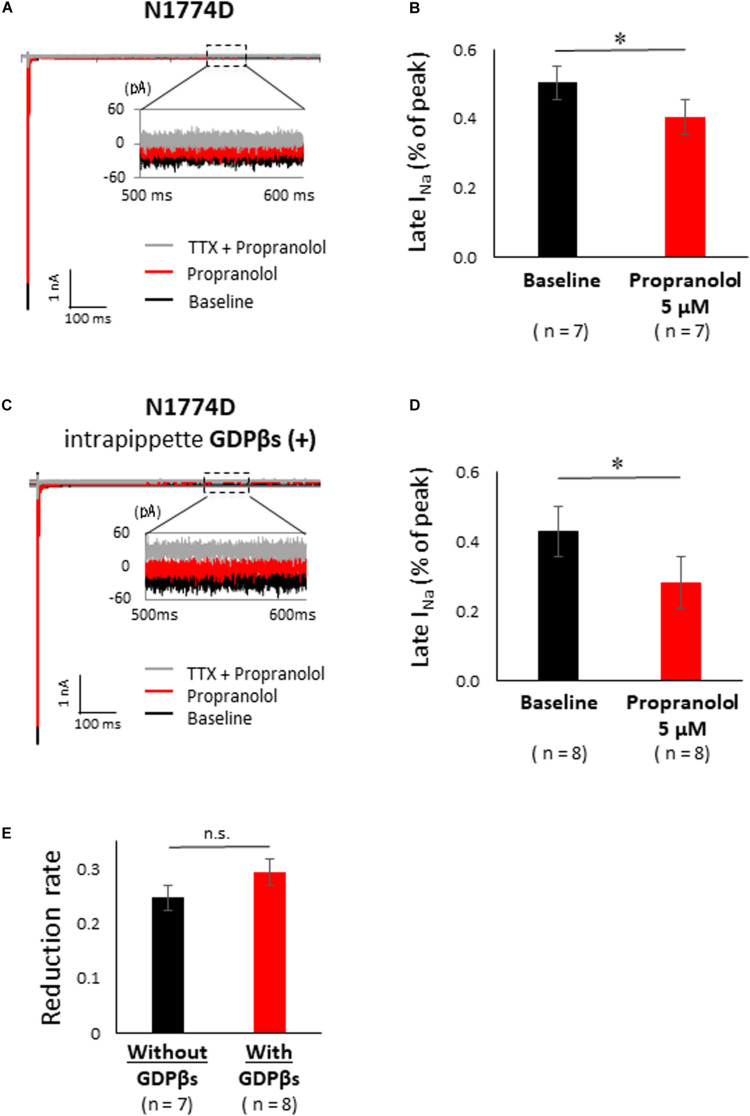
Effect of propranolol on late sodium current in N1774-hiPSC-CMs. **(A)** Typical late sodium current traces recorded at baseline (black line), after 5 μM propranolol application (red line), and after the additional treatment with 20 μM tetrodotoxin (TTX) (gray line). **(B)** Statistical analysis of the effect of propranolol on late sodium current. The data pooled from different clones in N1774D-hiPSC-CMs were analyzed. Late sodium current was normalized to peak sodium current. Propranolol significantly reduced the ratio of late/peak sodium current. ^∗^*p* < 0.001, vs. baseline. **(C)** Representative trace of late sodium current in the presence of intrapipette GDPβs. Black line is at baseline, red line is in the presence of 5 μM propranolol, and gray line is in the presence of 5 μM propranolol and 20 μM TTX. We recorded sodium currents in the presence of 5 μM propranolol following addition of 20 μM TTX after recording at baseline. **(D)** Summary of efficacy of propranolol on late sodium current after intrapipette GDPβs treatment. Propranolol significantly reduced the ratio of late/peak sodium current in the presence of intrapipette GDPβs. ^∗^*p* < 0.001, vs. baseline. **(E)** Reduction rate of late sodium current with and without GDPβs. There is no significant difference between in the absence and the presence of intrapipette GDPβs. GDPβs, guanosine diphosphate βs; TTX, tetrodotoxin.

Figure 5A shows the representative traces of late sodium current at baseline and after administration of 5 μM propranolol to N1774D-hiPSC-CMs. Propranolol treatment decreased the ratio of late/peak sodium current by approximately 25%, from 0.53 ± 0.05 to 0.40 ± 0.06% (*n* = 7; *p* < 0.001; Figure 5B). These results suggested that propranolol attenuated late sodium current and thus shortened APDs in N1774D-hiPSC-CMs.

### Propranolol Directly Inhibited the Late Sodium Current in N1774D-hiPSC-CMs

Finally, to examine whether propranolol affected the sodium channels through G protein cascade or not, we recorded late sodium currents by using intrapipette guanosine diphosphate βs (GDPβs), a G protein inhibitor (Figure 5C). The late sodium current was significantly reduced by treatment with propranolol and 1 mM intrapipette GDPβs (baseline, 0.43 ± 0.07% vs. propranolol 0.28 ± 0.07%, *n* = 8; *p* = 0.01; Figure 5D). However, the reduction rate of late sodium current by propranolol treatment was not statistically different after addition of intrapipette GDPβs (without GDPβs: 24.7 ± 4.8% vs. with GDPβs: 30.9 ± 6.9%; *p* = 0.48; Figure 5E). Thus, propranolol directly blocked sodium channels and did not affect them via G protein pathway through β-adrenergic receptors.

**FIGURE 6:**
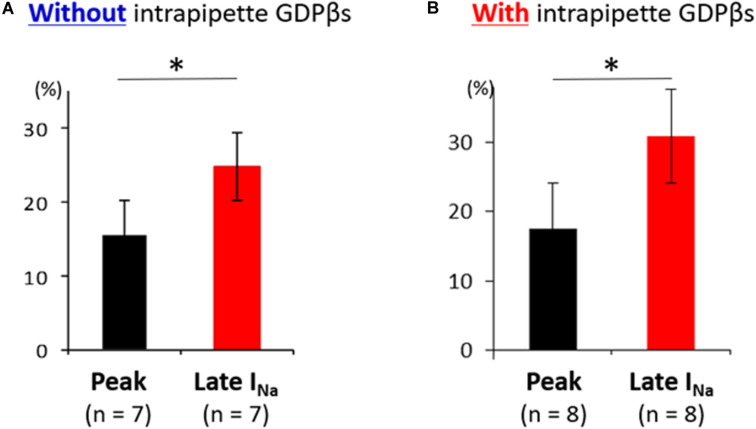
Effect of propranolol on peak and late sodium current in N1774D-hiPSC-CMs. Effect of propranolol on peak and late sodium current in the absence **(A)** and the presence of intrapipette GDPβs **(B)**. The data pooled from different clones in N1774D-hiPSC-CMs were analyzed. **(A)** Without intrapipette GDPβs, the rate of reduction was larger in late sodium current compared to in peak sodium current. **(B)** In the presence of intrapipette GDPβs, the rate of reduction was also larger in late sodium current compared to in peak sodium current. ^∗^*p* < 0.001, vs. peak sodium current.

Additionally, the suppression of late sodium current by propranolol was larger than that of the peak current whether in absence or presence of intrapipette GDPβs (Figure 6). No significant difference in the suppression rate of the peak and the late sodium currents were observed whether in absence or presence of GDPβs (Figure 6).

## Discussion

### Patient-Specific iPSC-CMs as a Cell Model for Studying *SCN5A*-N1774D Associated LQT3

The efficacy of β-blockers has been controversial in patients with LQT3 (Moss et al., 2000; Schwartz et al., 2001; Priori et al., 2004). Recently, a large clinical study demonstrated the efficacy of β-blockers on patients with LQT3 (Wilde et al., 2016); however, the pharmacological mechanism of β-blockers in LQT3 remains unclear.

Recently, the iPSC technology enables us to analyze the disease-causing mechanism of inherited arrhythmia disorder using self-beating cardiomyocytes with the same genetic background as the patient, and several studies of hiPSC-based disease modeling of LQT3 have been reported (Davis et al., 2012; Fatima et al., 2013; Ma et al., 2013; Terrenoire et al., 2013; Spencer et al., 2014; Yoshinaga et al., 2019). To elucidate the mechanism, we established the LQT3-hiPSC-CM cell model from a patient carrying the *SCN5A*-N1774D mutation who was effectively treated with propranolol. In accordance with his clinical course, propranolol attenuated the increased of late sodium current and prolonged APDs in N1774D-hiPSC-CMs, compared to the control cells. In addition, by using GDPβs, an inhibitor of G proteins, we demonstrated that propranolol inhibited the late sodium current through a pathway other than β-adrenergic receptor signaling pathway. Thus, the efficacy and pharmacological mechanism of propranolol have never, to our knowledge, been hitherto elucidated in an LQT3-hiPSC-CM model.

In our previous report (Kato et al., 2014), we analyzed *SCN5A*-N1774D channels using a heterologous expression system in HEK 293 cells and determined that N1774D channels displayed increased peak sodium current densities (2.2 times), a hyperpolarized shift of steady-state activation curve by 7.9 mV, and increased late sodium current densities by 1.7 times, compared to those of wild-type channels. In our LQT3-hiPSC model, the steady-state activation curve was negatively shifted by 12.8 mV and the peak and late sodium current densities were 1.9 and 9.3 times greater, respectively, when compared to those of the control hiPSC-CMs (Figures 2, 3). Considering that our hiPSCs carried the heterozygous *SCN5A*-N1774D mutation, the electrophysiological properties of hiPSC-CMs showed similar trends but different degrees of change in sodium channel kinetics to those of the heterologous expression system, possibly it might result from the differences between the two cell types.

### β-Blocker Therapy for Patients With LQT3

β-blocker therapy is effective for treatment of patients especially with LQT1 and LQT2, who experience cardiac events after adrenergic stimulation, such as exercise or emotional stress. In LQT1 and LQT2, β-blockers clinically reduce the rate of cardiac events and mortality (Moss et al., 2000; Schwartz et al., 2001; Priori et al., 2004). Therefore, based on the clinical evidence, β-blocker therapy is recommended as the first-line treatment for LQT1 and LQT2 (Priori et al., 2013).

In contrast, Schwartz et al. (2001) reported that the recurrence of syncope and death rates in patients with LQT3 were higher (50% and 17%, respectively, *n* = 18) than those with LQT1 and 2. Additionally, Priori et al. (2004) reported that 9 out of 28 (32%) LQT3 patients experienced a cardiac event while on β-blocker therapy at a rate four-fold higher than that of LQT1 patients. Even though the number of LQT3 patients enrolled in the study was small, β-blocker therapy in LQT3 was suggested to be ineffective in preventing fatal arrhythmias and even contraindicated or harmful due to the clinical feature of LQT3, that cardiac events mainly occur at rest, during sleep or bradycardia.

However, recently, the efficacy of β-blocker therapy was analyzed in a large multicenter LQT3 cohort study (Wilde et al., 2016). Wilde et al. (2016) investigated 406 LQT3 patients and demonstrated that β-blocker therapy reduced the risk of life-threatening cardiac events in female patients >1 year of age. In the present study, our patient suffered from repetitive TdPs since birth and propranolol was definitely effective in suppressing those events. Despite accumulating clinical evidence that β-blocker therapy might be beneficial to LQT3 patients, the underlying mechanism by which β-blockers suppress malignant ventricular arrhythmias remains mostly unclear. In our LQT3-hiPSC model, prolonged APDs at baseline were attenuated after propranolol administration (Figure 5), which was consistent with the clinical efficacy of β-blocker therapy in LQT3.

### Why Are β-Blockers Effective in Patients With LQT3?

#### Cardiac Sodium Channel and β-Adrenergic Stimulation

Cardiac sodium channels are known to be modulated by β-adrenergic stimulation via phosphorylation sites on protein kinases A and C, PKA and PKC, respectively (Grant, 2009). In cardiac sodium channels, PKA activation increased the peak sodium current density and shifted both steady-state activation and inactivation to the hyperpolarized direction (Matsuda et al., 1992; Ono et al., 1993). On the contrary, PKC activation reduced peak sodium current densities and caused a negative shift of the steady-state inactivation curve (Valdivia et al., 2009).

PKA activation did not affect the late sodium current in wild-type sodium channels. On the other hand, in LQT3-related mutant sodium channels, PKA activation had little or no effect on Y1795C and Y1795H channels, but it enhanced the late sodium current in ΔKPQ, D1790G, R1623G, and V2016M channels (Chandra et al., 1999; Tateyama et al., 2003; Tsurugi et al., 2009; Chen et al., 2016).

In LQT3 mouse models, propranolol efficacy to prevent lethal arrhythmias has been contradictory. Fabritz et al. (2010) showed that chronic propranolol therapy did not suppress the carbachol-mediated ventricular arrhythmias, such as TdPs, in a heterozygous knock-in *SCN5A*-ΔKPQ LQT3 mice. On the other hand, Calvillo et al. (2014) reported that propranolol pretreatment prevented carbachol-mediated malignant ventricular tachyarrhythmias in the *SCN5A*-ΔKPQ LQT3 knock-in transgenic mice. Thus, the pharmacological mechanism of β-blockers in LQT3 mice and human cardiomyocytes derived from patients with LQT3 has remained uncertain until now. In the present study, we demonstrated that propranolol attenuated the prolonged APD, by reducing the late sodium current in the patient-derived LQT3-hiPSC-CMs, and provided new supportive evidence of its efficacy on LQT3 by using a human CM model.

#### Propranolol Has an Inhibitory Effect on Sodium Channels but Not Through β-Adrenergic Receptor Blockade in an LQT3-hiPSC Model

Propranolol was previously reported to have a unique blocking effect on sodium channels, which was not identified in other β-blockers (Wang et al., 2010). In a heterologous expression system using HEK293 cells, only propranolol, not metoprolol or nadolol, blocked the cardiac sodium channels similar to local anesthetics, and not by acting as β-blockers (Wang et al., 2010). They also demonstrated that the block depends on a critical D4/S6 residue, F1760, involved in local anesthetic effects. In addition, Bankston and Kass (2010) demonstrated that propranolol decreased late sodium current in F1473C and ΔKPQ mutant channels. In this study, we showed no significant difference in the suppression rate of late sodium current by propranolol in presence, or not, of intrapipette GDPβs, an inhibitor of G proteins (Figure 5E). This was found to be consistent with the previous reports that propranolol has a sodium channel blocking effect through interfering with the cascade other than G protein cascades (Bankston and Kass, 2010; Wang et al., 2010).

Interestingly, Bankston and Kass (2010) also reported that propranolol within 40 nM to 40 μM range preferential inhibited late but not peak sodium currents. In this study, administration of 5 μM propranolol reduced late sodium current, but it slightly decreased peak sodium current (Figure 6 and Table 3). Regarding the plasma concentration of propranolol, it was reported that there was wide inter-individual variation from 0.04 to 4 μM in peak plasma concentrations, and an increase in the dose up to plasma levels of approximately 0.4 μM was suggested when sufficient therapeutic effects were not achieved (Woosley et al., 1979). Unfortunately, the plasma concentration of propranolol in the patient in this study was not available, and we recorded APs and late sodium current with only 5 μM propranolol using hiPSC-CM model. In addition, we did not examine the effect of propranolol on the kinetics of sodium channel current, which was demonstrated in a heterologous expression system (Wang et al., 2010). Therefore, further examination is needed to reveal the detailed relationship between propranolol dose and the effects on electrophysiological properties in hiPSC-CMs.

**TABLE 3:** The change of peak and late sodium current after the administration of propranolol in N1774D-hiPSC-CMs.

	Without GDPβs			With GDPβs			
	Baseline (pA)	Propranolol 5 μM (pA)	Reduction rate (%)	Baseline (pA)	Propranolol 5 μM (pA)	Reduction rate (%)	*p* value
Peak current	−11627 ± 2177	−9857 ± 1933	15.6 ± 4.6	−7779 ± 1035	−6599 ± 1044	17.5 ± 3.3	0.74
Late current	61.9 ± 15.7	37.5 ± 8.3	24.7 ± 4.8	38.0 ± 8.6	19.3 ± 4.5	30.9 ± 6.9	0.48

*Data are expressed as means ± standard error of measurement. *p* value for vs. with GDPβs in reduction rate. The data pooled from different clones in N1774D-hiPSC-CMs were analyzed.*

### Study Limitations

In the present study, we did not employ the isogenic control line which will prove the phenotype is directly imputable to the mutation. In addition, we did not examine the efficacy of other β-blockers. Another limitation is that hiPSC-CM is an *in vitro* cellular model; therefore, it is impossible to assess the role of sympathetic nervous system. Clinically, left cardiac sympathetic denervation was reported to inhibit cardiac events in LQT3 patients (Schwartz et al., 2004), which indicated that β-blockers would have an antiarrhythmic effect by suppressing sympathetic nervous system in LQT3.

### Conclusion

We successfully recapitulated the LQT3 disease phenotype in the *SCN5A*-N1774D-hiPSC-CMs. Additionally, the propranolol efficacy and mode of action were demonstrated in this cell model. Specifically, propranolol blocked sodium channels by means of a unique mechanism unrelated to β-adrenergic signaling pathway, by preferentially inhibiting late rather than peak sodium current.

## Data Availability Statement

The datasets generated for this study are available on request to the corresponding author.

## Ethics Statement

The studies involving human participants were reviewed and approved by the Ethics Committee of Kyoto University. Written informed consent to participate in this study was provided by the participants’ legal guardian/next of kin. The animal study was reviewed and approved by Ethics Committee of Kyoto University.

## Author Contributions

SH, TM, and MHo: conceptualization. SH and TM: methodology and writing – original draft. SH, TM, DM, JW, YYa, YW, FY, SN, TH, MHa, HK, JG, AK, MN, JY, and KC: investigation. TM, DM, MHa, and MHo: writing – review and editing. TM and MHo: funding acquisition. TM, SO, YYo, and MHo: resources. DM, SO, YYo, MHo, and TK: supervision. All authors contributed to the article and approved the submitted version.

## Conflict of Interest

YYo owns stock in iPS Portal. The remaining authors declare that the research was conducted in the absence of any commercial or financial relationships that could be construed as a potential conflict of interest.
